# Inflammation and autoimmune indicators in the differential diagnosis of autism spectrum disorders in children

**DOI:** 10.1192/j.eurpsy.2022.925

**Published:** 2022-09-01

**Authors:** L. Androsova, N. Simashkova, S. Zozulya, O. Shushpanova, I. Otman, T. Klyushnik

**Affiliations:** 1 Mental Health Research Centre, Laboratory Of Neuroimmunology, Moscow, Russian Federation; 2 Mental Health Research Centre, Department Of Childhood Psychiatry, Moscow, Russian Federation

**Keywords:** autism spectrum disorders, differential diagnosis, inflammation amd autoimmune markers

## Abstract

**Introduction:**

Autism spectrum disorders (ASD) is one of the most urgent problems of psychiatry because of their high prevalence, diagnostic difficulties as well as insufficient knowledge of the pathogenetic mechanisms.

**Objectives:**

To determine the number of inflammation markers in patients with various forms of ASD in links with features of a clinical condition for creating diagnostic criteria for differential diagnosis and improve reliability.

**Methods:**

The clinical examination of patients (135 children with various ASD forms) was carried out by using psychometric scales (CARS, BFCRS, CGI-S). The activity of inflammation markers (LE and α1-PI) and the level of autoantibodies to S-100b and MBP were measured in plasma. Complex evaluation of immune system activation was also conducted, taking into consideration interactions of innate and adaptive immunity.

**Results:**

Non-psychotic ASD forms (Asperger’s syndrome and Kanner’s syndrome) were not accompanied by a change of the immunological indices in comparison with control. In psychotic ASD forms, a significant increase of the studied indices was revealed (р<0.05). Correlation between the complex evaluation of the immune system activation and the stage of the disease (r=0.49, р<0.05) was demonstrated. Also the significant correlations between the severity of autistic disorders according to CARS (r=0.48, p<0.05), catatonic disorders by BFCRS (r=0.42, p<0.05), and the assessment by CGI (r= 0.61, p<0.05) were observed.

**Conclusions:**

The immune markers as well as their complex evaluation may be used as additional diagnostic criteria in the clinical examination for differential ASD diagnostics and assessment of the quality of remission, and also monitoring of the patient condition.

**Disclosure:**

No significant relationships.

